# Human Tissue in the Evaluation of Safety and Efficacy of New Medicines: A Viable Alternative to Animal Models?

**DOI:** 10.5402/2011/806789

**Published:** 2011-07-06

**Authors:** Robert A. Coleman

**Affiliations:** 27 Wodehouse Terrace, Falmouth, Cornwall TR11 3EN, UK

## Abstract

The pharma Industry's ability to develop safe and effective new drugs to market is in serious decline. Arguably, a major contributor to this is the Industry's extensive reliance on nonhuman biology-based test methods to determine potential safety and efficacy, objective analysis of which reveals poor predictive value. An obvious alternative approach is to use human-based tests, but only if they are available, practical, and effective. While *in vivo* (phase 0 microdosing with high sensitivity mass spectroscopy) and *in silico* (using established human biological data), technologies are increasingly being used, *in vitro* human approaches are more rarely employed. However, not only are increasingly sophisticated *in vitro* test methods now available or under development, but the basic ethically approved infrastructure through which human cells and tissues may be acquired is established. Along with clinical microdosing and *in silico* approaches, more effective access to and use of human cells and tissues *in vitro* provide exciting and potentially more effective opportunities for the assessment of safety and efficacy of new medicines.

## 1. Introduction

**Figure 1:**
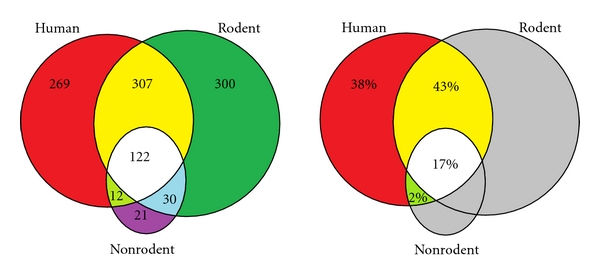
Venn diagrams of compounds causing adverse effects in the livers of humans, rodents, and/or nonrodents. (a) The numbers of compounds causing effects in each species class alone and in more than one class, and (b) the proportions of compounds reporting liver effects in humans that have effects only in humans, in humans and rodents, in humans and nonrodents, and in all three species classes, as defined by assertions derived from Medline in a total of 1061 compounds (see http://www.biowisdom.com/downloads/SIP_Board_Species_Concordance.pdf).

It is generally agreed that the pharma industry has a problem in bringing safe and effective new drugs to market. This may well be due, at least in part, to the overreliance of the industry on using animals as human surrogates, an issue that has been of concern to many working in the area for decades [1–6]. Indeed, the most widely used animal species, rodents, dogs, and even nonhuman primates, have all been shown to be unreliable in their ability to predict drug behaviour in man. A comparison of the bioavailability of a range of drugs in man with that in these three species by Grass and Sinko demonstrated a very poor level of correlation [7]. Furthermore, the retrospective study by Olson and colleagues [6] showed that for some systems, the predictive value of animal studies to identify potential toxicity in human subjects performed little better than the spin of a coin. Interestingly, Olson's findings correlate rather well with those of Fletcher [3], published more than 20 years earlier. Further support has been generated in a study on species concordance for liver injury [8] using a safety intelligence programme drawing on data in Medline and EMEA European Public Assessment Reports (EPAR). In a range of more than 800 (Medline) and 130 (EPAR) marketed and withdrawn compounds with evidence of liver toxicity in man, only 60% (Medline) and 49% (EPAR) proved similarly toxic in rodents, and only 17% and 35% in both rodent and nonrodent experimental species (Figure 1). In the light of such questionable predictive power, it seems surprising that such store is still set by animal safety data. While this has always been the case, concern has been expressed that animal shortcomings are set to become ever greater with the increased focus on human-targeted biologicals [9]. There is a strong case, therefore, to look more critically at the current methods used to indicate the potential safety and efficacy of new drugs and to explore whether there are better ways of doing it.

## 2. The Role of Animals in Safety and Efficacy Testing

It appears still to be widely believed that despite their acknowledged shortcomings, animal studies are pivotal in drug discovery, and it has been stated that “virtually every medical achievement of the last century has depended directly or indirectly on research with animals” [10–12]. While a powerful statement, it is one that has spurious justification. In support, as far as new medicines are concerned, it is undeniable that they will all have been tested in animals and that these tests will have declared the compounds both sufficiently safe and effective for evaluation in man. This is so because the industry requires that drugs are demonstrably effective in their animal models before they will advance those drugs to the clinical stage. And as to safety, a sufficiently blemish-free profile in experimental animals is a mandatory aspect of the regulatory approval process. So, while it is true that all drugs have been tested and judged safe and effective in animals, it is not clear to what degree this is relevant to their profiles in human subjects. Indeed, if it were decreed that only compounds coloured yellow or smelling of roses could advance to clinical testing, then all successful drugs would have these characteristics, but it would be absurd to suggest that these properties were essential to the identification of safe and effective new medicines. What the current confidence in animal prediction ignores is firstly the fact that the large majority of drugs entering clinical trials are found either to lack clinical efficacy or to cause either undesirable side effects or frank toxicity and secondly that we have no idea how many potentially valuable medicines have been committed to the dustbin on the basis of spurious animal data. 

To put the role of animal surrogates for human safety in perspective, it is interesting to consider what would happen if safety in experimental animals was required for the approval of foodstuffs for human consumption. If this was the case, we would not have avocados, blue cheese, Brussels sprouts, cabbage, chocolate, coffee, garlic, grapes, liquorice, onions, or many other common and demonstrably safe foodstuffs [13, 14], as all have proved to be poorly tolerated or even toxic in rodents and/or dogs. And more to the point, the recent experience with the Te Genero drug, TGN1412 [15] that caused such devastating effects in human volunteers at a dose 500-fold lower than that well-tolerated by nonhuman primates, illustrates the shortcomings of safety assessment in animals, particularly in the case of agents specifically designed to interact with human targets, as are an increasing number of new biological medicines. 

The situation is no better as far as predictions of efficacy are concerned. Cancer is a particularly good example, where mouse models abound but have a very poor record in predicting efficacy in man [16–19]. It has been generally accepted that approximately 95% of novel cancer drugs, effective in animal models, have proved to be ineffective in the clinic [20, 21]. And if one looks at the current armoury of antiasthma treatments, primarily corticosteroids, beta-agonists, theophylline, cromones, and antileukotrienes, only the latter can claim that the original discovery and development of the class was based on animal experiments. Conversely, if we review compounds promoted as potential new treatments for asthma based on studies in mice, guinea pigs, and sheep, we see, among others, antihistamines, antagonists at neurokinin, bradykinin, PAF, thromboxane and endothelin receptors, calcium channel blocking drugs, potassium channel openers, statins, and PPAR gamma agonists, none of which has ultimately proven clinically useful [22–24]. 

**Figure 2:**
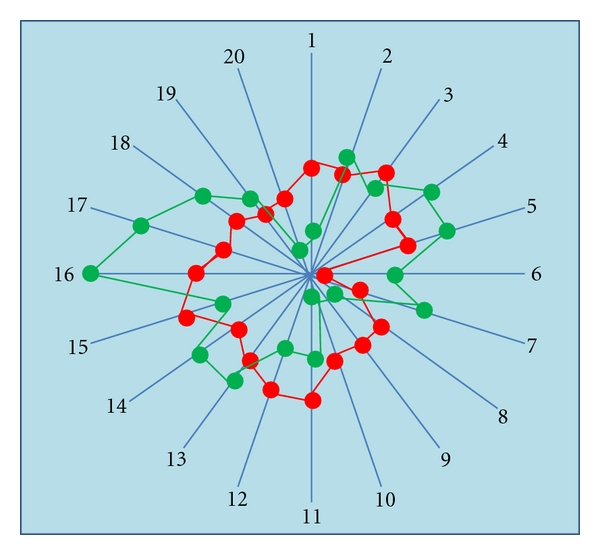
Comparison of QRT-PCR expression patterns for 5-HT_2B_ in 20 tissues from mouse (red) and human (blue). Each numbered radial arm represents a different tissue type, and concentric circles represent magnitude of gene expression in mRNA copy number per 100 ng total RNA. Data points are mean values from 3 independent values (i.e., generated from 3 samples of each tissue type, each obtained from a separate animal/donor). Tissues are (1) heart, (2) oesophagus, (3) stomach, (4) jejunum, (5) colon, (6) pancreas, (7) liver, (8) cerebellum, (9) frontal cortex, (10) spinal cord, (11) trachea, (12) lung parenchyma, (13) kidney, (14) bladder, (15) ovary, (16) uterus, (17) vas deferens, (18) testis, (19) spleen, (20) skin. (see Coleman, [26]).

The standing of the mouse as an experimental human surrogate rose considerably with the completion of the mouse and human genomes and the realisation of the fundamental genomic similarity of the two species. According to Home Office statistics in 2008 in the UK, more than 2 million mice were used in scheduled procedures, and this represented over 60% of the total number of animals used [25]. A study of gene expression in mouse and man reveals many critical differences: to take a specific example, it has been demonstrated that the patterns of body-wide expression of 5-HT_2b_ receptor mRNA in the two species are quite different [26] (Figure 2). Furthermore, there is only an 82% concordance in the sequences of the genes encoding this receptor in these two species [27]. And what is even more important, a study of the affinity of this receptor to its natural ligand, 5-HT, revealed that the avidity of the mouse receptor for 5-HT is at least 100-fold lower than that of the human receptor for the hormone [27]. With such a difference, it is inconceivable that the 5-HT_2b_ receptor in these two species serves the same role. 

This is not to say that all animal tests are valueless; for some classes of drug, particular animal tests have proved highly predictive of clinical efficacy and/or safety. However, this is patently not a general rule, and validation or otherwise is only achieved with the benefit of hindsight, providing a rather insecure basis for assessing novel chemical entities. It is undoubtedly timely, therefore, to question the continued use of animal surrogates on the basis of both ethics and logic. This is a view supported by a number of critical publications, including that of [28], which showed that the likelihood of animals predicting human clinical outcome was not significantly better than 50 : 50. So, if animals' performance in this regard is so poor, why do we continue to use them? There are a number of reasons, some of these relating simply to the way things have always been done, the insistence of the regulators for animal data, and of course the difficulty in finding an alternative.

## 3. Are Nonanimal Alternatives Possible?

The obvious alternative is to concentrate on human, rather than nonhuman, biology in preclinical testing. But how? The three approaches available to the drug discovery scientist are *in vivo*, *in silico,* and *in vitro*. 

A number of noninvasive techniques have been developed to evaluate drug activity in human subjects, such as CAT, MRI, PET, and SPECT scans, transcranial magnetic stimulation (TMS), and laser Doppler perfusion imaging although the value of these for determining potential safety of new medicines is not clear. However, there is increasing interest in the use of microdosing (i.e., administration of doses some 100-fold lower than the lowest intended clinical dose), and there is a growing body of evidence that it has predictive value [29–31]. There is little doubt that if the early encouraging data are replicated, this approach will be increasingly used in future drug testing. However, this approach is primarily of value in exploring the likely pharmacokinetic fate of new drugs and provides limited information as to efficacy or safety although it can provide an early indication of the likely generation of potentially hazardous or indeed efficacious metabolites. It is relevant at this stage to mention drug testing in brain-dead individuals, a model that has been conducted continuously, albeit at a low level for over 25 years [32]. This approach, which technically has a lot to commend it, uniquely permitting the generation of highly relevant data, raises a number of ethical issues, which are beyond the scope of the present paper, and which I will leave to others to debate [33]. 

In contrast, *in silico* testing raises no such ethical issues, and is becoming increasingly accepted as a part of the preclinical profiling of new drugs. *In silico* testing, using computational approaches, is showing promise although at present, it is generally recommended that a consensus of indicators from a variety of different models be used, rather than relying on a single model. However, with many models already commercially available, the reliability of this approach to predicting drug behaviour in humans will undoubtedly grow, and as a result of the European REACH Directive [34], it is now supported by the OpenTox program [35]. However, this is always likely to remain a supportive element, rather than a primary indicator. 

It is clear that such *in vivo* and *in silico* approaches are increasingly accepted as key contributors to today's drug development programs, and as such, they will not be dealt with further in this paper. The area that is really being sorely neglected is the use of human *in vitro* techniques. The value of the *in vitro* approach is nicely illustrated by TGN1412, where following its disastrous clinical trial [15], an *in vitro* method was rapidly developed that modelled the potentially fatal cytokine storm experienced by the clinical volunteers [36, 37]. Had this been developed and used before exposing human subjects to the drug, the trial would never have taken place. Surely, the time has come for there to be a rigorous prospective evaluation of human-based approaches, not only* in vivo *and *in silico*, but critically also *in vitro*, as alternatives to the deeply flawed, animal-based approaches in current use in the identification of potential safety issues for new drugs in man.

### 3.1. Human Biology-Based Methods

Despite the establishment in 1959 of Russell and Burch's principle of the 3Rs [38], the introduction of nonanimal tests for safety has been painfully slow, and of human-based tests even slower. It was not until the 1970s, when Ames et al. introduced his bacterial mutagenicity test [39], that the first nonanimal test was made a regulatory requirement. However, since Ames, there have been few other nonanimal tests that have achieved regulatory accreditation and those that have are limited largely to dermal toxicity and mutagenicity testing [40, 41]. However, there are a few animal cell/tissue tests [42] that could theoretically utilise the corresponding human material, but in the main, *in vivo* animal tests remain the basis of the bulk of regulatory required safety testing. Interestingly, a human cell-based test has now been developed to replace the Ames Test [43], but it has yet to be granted regulatory accreditation. Exactly why more effort is not being put towards developing more human-based test systems is not clear, but it does appear to be something of a vicious circle, with both industry and the regulatory authorities waiting for the other to make the first move. But what is certain is that the regulators will only approve any approach when there is convincing demonstration of value, so it is clearly down to industry to take up the challenge. 

Much use may be made of human isolated cells and tissues in supporting pharmaceutical R&D through the application of relatively straightforward *in vitro* assays, such as blood cells, hepatocytes, pancreatic islets, and various smooth muscle preparations, but one of the prime objections to adoption of *in vitro* human tissue models is that it is impossible to adequately model the complexity of the whole body in isolated tissues. While this argument undoubtedly has some validity, it is too easy simply to say “it cannot be done”, a position that seriously undervalues human ingenuity when faced with a seemingly intractable problem. Indeed, with the development of powerful new technologies, such modelling may be nearer to realisation than is generally appreciated. The ability of scientists to model complex pathological processes using a combination of simple assays is illustrated by the apparently successful method of predicting nausea and vomiting in man using a range of approaches including the use of human cells [44]. 

**Figure 3:**
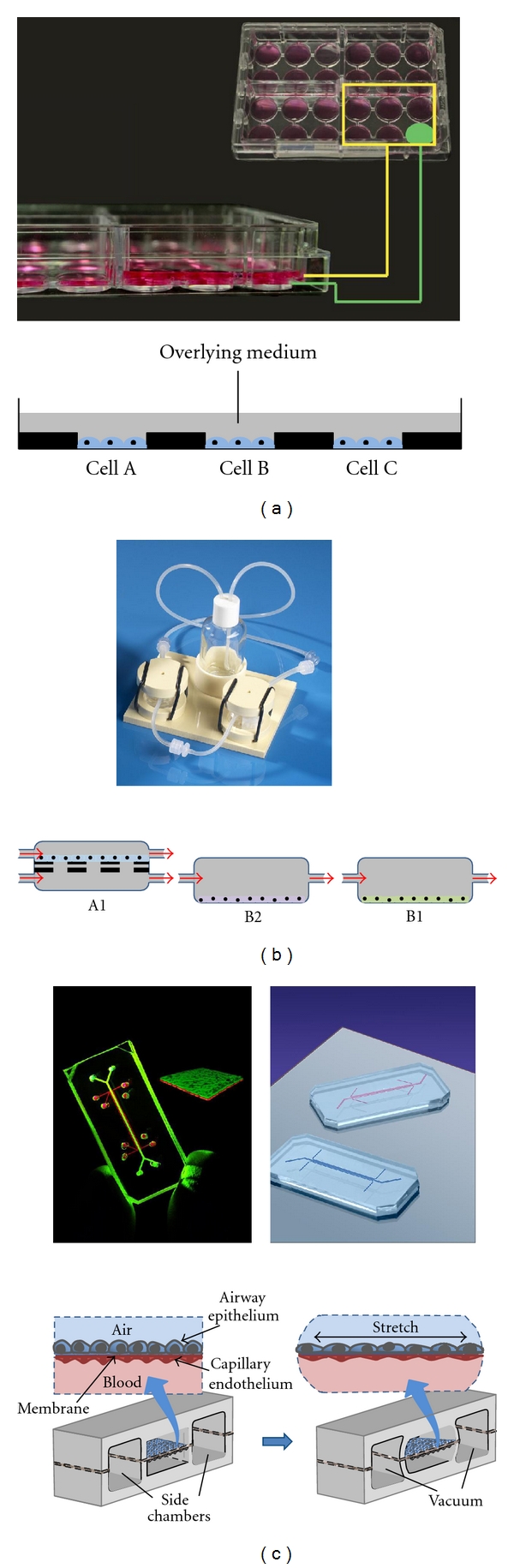
Some examples of tests involving cell cocultures. Such methods allow simultaneous application of compounds to multiple cell types, means of studying the influence (e.g., via secreted factors) of one cell type over the other, and of the effects of metabolites produced by one cell type on the function of another. (a) IdMOC technology. Typically, cells are seeded in inner wells (marked in green) and incubated for 24 hours to allow attachment, after which the larger, rectangular (yellow), well is flooded with media containing substrates or test compounds. The flooding medium permits interconnection of multiple inner wells cell mimicking the integration of multiple organs via the systemic circulation. (b) Upper panel shows quasivivo system, lower panel a schematic showing how chambers can be connected in series with different cell types in each. The first chamber A1 is a dual flow chamber with different liquids/media on either side of a porous membrane or scaffold on which cells are being cultured. The A1 type of chamber can be adapted to provide an air-liquid interface by substituting one of the liquid flows by air. (c) Lung on a chip. Upper panel shows the chips, lower panel a schematic illustrating the detail of the design and the arrangement of bronchial epithelial and airway vascular endothelium either side of a porous membrane. Chips are 2 cm—long polymer devices designed to mimic the function of the human lung. The microfluidics system incorporates an alveolar-capillary interface that is flanked by two side chambers. The alveolar-capillary interface consists of a porous, flexible, 10 *μ*m—thick polymer membrane coated with extracellular matrix (ECM) that separates a channel containing human alveolar epithelial cells and a layer of air from a channel containing human pulmonary microvascular endothelial cells and a flowing layer of cell culture media. Application of vacuum to the side chambers deforms the thin walls separating those chambers from the interface, causing the flexible polymer membrane to stretch—thus mimicking the mechanical effects of breathing.

The answer almost certainly lies not only in considering drug actions on cell types in isolation, but also through the integration of a range of technological approaches applied to human tissues and cells under conditions that better reflect the cell : cell, tissue : tissue, and even organ : organ interactions that are operational in the human body. Such an integrated *in vitro* approach may be regarded as “*proxi-vivo*”. There have been considerable advances in the development of such constructs. This is particularly important in considering the effects not only of the drugs themselves, but also of their metabolic products, and these are likely to be generated by tissue(s) other than that in which an adverse effect may originate. Such integrated modelling is being provided in a range of different forms (Figure 3), for example, using microfluidics [45, 46], the so-called quasi-vivo multicompartmental modular system [47], or wells within wells [48], all of which allow drugs to be exposed to key tissues in a fashion approximating that *in vivo*. Such approaches allow for example the exposure of a drug to liver cells prior to contact with cells of a target organ(s), allowing an understanding of the activity not only of the drug itself, but also of its metabolic products, more closely mimicking what is likely to occur *in vivo*. An impressive example of this approach is the “lung on a chip” developed by Harvard scientists [49]. If this model proves useful, it will undoubtedly eventually be succeeded by “gut on a chip”, “cardiovascular system on a chip” and many others. Such coculture has also been used to establish a model of synovial fibroblast-induced cartilage destruction as a model of rheumatoid arthritis [50]. 

There are other ways in which pathophysiologically relevant cell interactions may be incorporated in an *in vitro* assay. One interesting approach involves exposing pathologically relevant combinations of cell types to various different challenges, and measuring the release of a wide panel of gene products [51]. This has shown that the influence of test drugs on this pattern of released products can be indicative of the test compound's biological mechanism of action. The use of various analyses of correlation and clustering in comparison to an extensive reference set allows considerable insight into both therapeutic and pathological aspects of the biological profile of novel compounds. Similar approaches have been developed by other companies, but using other markers of biological activity, for example, gene expression [52], transcription factors [53] and microRNAs [54]. Such technologies represent hypothesis-free approaches more akin to *in vivo *safety testing than traditional *in vitro* assays in which a compound is commonly assayed against a particular target in a particular tissue or cell type. 

While cell culture systems can be rightly criticised for their generally nonphysiological nature, particularly in regard to their limited cell number, inadequate perfusion, and the existence of edge effects, considerable efforts have gone into improving on this, with the use of tissue slices, scaffolds and other culture support, and also 3D culture methods [55–57]. The achievement of more physiological 3-dimensional cultures involving combinations of relevant cell types will undoubtedly represent a significant step towards more effective *in vitro* modelling of *in vivo* systems.

Another important source of human biological material is the stem cell. As our understanding of stem cells and the factors determining their differentiation grows, they will undoubtedly prove increasingly useful in the generation of model constructs for the testing of new drugs for both efficacy and toxicity [58]. Much work is already underway under the umbrella of the Stem Cells for Safer Medicines group [59], whose stated aim is *“To enable the creation of a bank of stem cells, open protocols and standardised systems in stem cell technology that will enable consistent differentiation of stem cells into stable homogenous populations of particular cell types, with physiologically relevant phenotypes suitable for toxicology testing in high throughput platforms.”* Indeed, as an example, stem cells have already been used to generate cardiomyocyte-like cells that can be used to model not just cardiac ion channel function or QT interval prolongation, but the potential to induce ventricular arrhythmias associated with torsade de pointes [60].

The value of any *in vitro* cell/tissue construct is of course only as great as the methods applied to detect drug activities. The answer to better assessment of drug effects, both in terms of potential efficacy and safety probably lies in an association of appropriate coculture systems along with improved high content type methods of detecting biological activity. Such approaches combined with modern clustering analysis and pattern recognition software will enable identification of activities undetectable by more conventional *in vitro* methods [61–63]. Such approaches are currently incorporated in the US EPA's ToxCast programme, which is specifically designed to identify better ways of identifying human safety both for environmental chemicals and more recently for pharmaceuticals [64].

There is no intention to suggest that at present we are in a position to simply switch from the current, largely animal-based system of safety testing to a more human-focused battery of tests. But on the basis of the considerable developments made in recent years, the onus now is on the biopharma industry and academia to apply themselves to the task of exploiting the ever-increasing range of human tissue-based technologies to develop more relevant and predictive methods of establishing the safety and efficacy of new medicines, and for government and the regulatory authorities to provide all the necessary encouragement.

It is highly likely that there will prove to be toxicities and disorders for which isolated cells and tissues do not, and may never, provide the whole answer, and where some reliance on experimental animals must remain. In such cases, however, comparative studies of relevant cells, tissues and associated pathophysiological processes in man and the chosen animal species should be undertaken to establish the relevance of the proposed animal model, before valuable resource is potentially wasted in the simple hope that the results will have clinical relevance.

## 4. Access to Human Biological Materials

While the attraction of human *in vitro* studies is in the wide range of functions that can be studied, it must be acknowledged that unless we improve radically our access to viable human tissues, such testing will represent a considerable bottleneck in preclinical drug testing programmes. At present in the UK we are limited almost exclusively to acquisition of tissues from surgery and *post mortem*. While tissue acquired from these sources is of considerable value, it is not enough. From these sources, we are limited in terms of the range of tissue types, the quantity that may be supplied, the quality and the frequency. These are issues that must be addressed if human tissue is ever to become a key component of preclinical efficacy and safety testing. I would suggest that the answer lies in access to tissues from both heartbeating and nonheartbeating organ donors. In the UK alone, there are more than 17 million people currently on the transplant donor register, and in the year between 1 April 2009 and 31 March 2010, over 3,700 organ transplants were performed [65]. If each of the donors of those organs had additionally donated nontransplantable organs/tissues for research, a vast amount of human-based research would have been possible. And this is potentially only the tip of the iceberg, as the UK Department of Health is currently striving to increase the availability of organs for transplant through the institution of NHS Blood and Transplant, and it would be hugely valuable if all organ retrieval for transplantation could be associated with additional retrieval of material for research.

But for human tissue research to be adopted as a key component of the drug testing paradigm, it must become a “need to have”, and that will only happen if it is demanded as a regulatory requirement. This will not happen until we can guarantee access to tissues of the required range, of appropriate condition, in sufficient quantity and with the necessary frequency. Collaboration between NHS Blood and Transplant and the pharma industry, together with buy-in by the general public to facilitate the availability and use of human tissue for research is essential to realise the full potential of a human-based approach to drug discovery and development.
